# Cost-effectiveness of a stepwise cardiometabolic disease prevention program: results of a randomized controlled trial in primary care

**DOI:** 10.1186/s12916-021-01933-6

**Published:** 2021-03-11

**Authors:** Daphne M. Stol, Eelco A. B. Over, Ilse F. Badenbroek, Monika Hollander, Mark M. J. Nielen, Roderik A. Kraaijenhagen, François G. Schellevis, Niek J. de Wit, G. Ardine de Wit

**Affiliations:** 1 Julius Center for Health Sciences and Primary Care, University Medical Center Utrecht, Utrecht, The Netherlands; 2 Netherlands Institute for Health Services Research (NIVEL), Utrecht, The Netherlands; 3 National Institute for Public Health and the Environment (RIVM), Bilthoven, The Netherlands; 4 Netherlands Institute for Prevention and E-health Development (NIPED), Amsterdam, The Netherlands; 5 Department of General Practice and Elderly Care Medicine, Amsterdam Public Health Research Institute, Amsterdam University Medical Centers (location VUmc), Amsterdam, The Netherlands

**Keywords:** Cardiometabolic diseases, Cardiovascular disease, Diabetes type 2, Economic evaluation, Cost-effectiveness analysis, Prevention, Primary care, Modelling study

## Abstract

**Background:**

Cardiometabolic diseases (CMD) are the major cause of death worldwide and are associated with a lower quality of life and high healthcare costs. To prevent a further rise in CMD and related healthcare costs, early detection and adequate management of individuals at risk could be an effective preventive strategy. The objective of this study was to determine long-term cost-effectiveness of stepwise CMD risk assessment followed by individualized treatment if indicated compared to care as usual. A computer-based simulation model was used to project long-term health benefits and cost-effectiveness, assuming the prevention program was implemented in Dutch primary care.

**Methods:**

A randomized controlled trial in a primary care setting in which 1934 participants aged 45–70 years without recorded CMD or CMD risk factors participated. The intervention group was invited for stepwise CMD risk assessment through a risk score (step 1), additional risk assessment at the practice in case of increased risk (step 2) and individualized follow-up treatment if indicated (step 3). The control group was not invited for risk assessment, but completed a health questionnaire. Results of the effectiveness analysis on systolic blood pressure (− 2.26 mmHg; 95% CI − 4.01: − 0.51) and total cholesterol (− 0.15 mmol/l; 95% CI − 0.23: − 0.07) were used in this analysis. Outcome measures were the costs and benefits after 1-year follow-up and long-term (60 years) cost-effectiveness of stepwise CMD risk assessment compared to no assessment. A computer-based simulation model was used that included data on disability weights associated with age and disease outcomes related to CMD. Analyses were performed taking a healthcare perspective.

**Results:**

After 1 year, the average costs in the intervention group were 260 Euro higher than in the control group and differences were mainly driven by healthcare costs. No meaningful change was found in EQ 5D-based quality of life between the intervention and control groups after 1-year follow-up (− 0.0154; 95% CI − 0.029: 0.004). After 60 years, cumulative costs of the intervention were 41.4 million Euro and 135 quality-adjusted life years (QALY) were gained. Despite improvements in blood pressure and cholesterol, the intervention was not cost-effective (ICER of 306,000 Euro/QALY after 60 years). Scenario analyses did not allow for a change in conclusions with regard to cost-effectiveness of the intervention.

**Conclusions:**

Implementation of this primary care-based CMD prevention program is not cost-effective in the long term. Implementation of this program in primary care cannot be recommended.

**Trial registration:**

Dutch Trial Register NTR4277, registered on 26 November 2013

**Supplementary Information:**

The online version contains supplementary material available at 10.1186/s12916-021-01933-6.

## Background

The increasing burden of cardiometabolic diseases (CMD), defined as cardiovascular disease (CVD), diabetes type 2 (DM2) and chronic kidney disease (CKD), is mainly caused by an unhealthy lifestyle and ageing. CMD are the major cause of death worldwide and associated with a lower quality of life and high healthcare costs [1]. In the Netherlands, CMD account for about one sixth of total Dutch healthcare costs [2]. CMD share common modifiable risk factors such as smoking, obesity, hypertension and hypercholesterolemia [3] and could be prevented by changes in lifestyle or pharmacological treatment [4].

To prevent a further rise in CMD and related healthcare costs, early detection and adequate management of individuals at risks could be an effective preventive strategy. European guidelines [4, 5] underline the importance of risk assessment and management of individuals without pre-existing CMD and CMD risk factors. In the Netherlands, the Dutch College of General Practitioners developed the “prevention consultation” guideline in 2011, which is a primary care-based stepwise CMD prevention program [6].

The effectiveness of early detection of CMD on long-term CMD morbidity and mortality is often questioned and the cost-effectiveness of these programs has not yet been established [7–11]. There is a lack of studies with robust economic evaluations alongside trials [11]. A recent review of Hilligsman and colleagues on the cost-effectiveness of early detection of CMD suggests that screening programs for DM2 and CVD could represent good value for money [10], although the heterogeneity between studies made an unequivocal conclusion difficult.

In 2013, the INTEGRATE study was designed to investigate the effectiveness and cost-effectiveness of a primary care-based stepwise CMD prevention program. The effectiveness analysis showed promising results on surrogate outcomes such as blood pressure and cholesterol levels with a significant decrease after 1 year of treatment [12]. However, given that implementation of structured CMD prevention in primary care is time and resource intensive, establishing long-term cost-effectiveness in terms of morbidity and mortality is required to justify widespread implementation.

Therefore, the aim of the present study was to describe costs and benefits after 1 year and to determine long-term cost-effectiveness of the CMD prevention program. In order to investigate this, we used a computer-based simulation model to project long-term health benefits and cost-effectiveness, assuming the prevention program was implemented in Dutch primary care.

## Methods

### Design

The INTEGRATE study (Dutch Trial Register number NTR4277) is a randomized controlled trial (RCT). In total, 37 Dutch general practices participated from April 2014 to April 2017. Stepwise CMD risk assessment followed by tailored lifestyle and/or pharmacological treatment, if indicated, was compared with care as usual. The control group was invited for the intervention 1 year later. Details about the study design, randomization, intervention components and measurements are described elsewhere [13].

### Participants

Eligible for participation were all patients listed in the participating practices aged 45–70 years without recorded CMD, hypertension and hypercholesterolemia and without antihypertensive, lipid-lowering or antidiabetic drugs according to the patient’s electronic health record (EHR) (see Additional file 1: flowchart 1).

### Intervention

Intervention participants were invited for a stepwise CMD prevention program. To identify high-risk patients—as the first step of the program—participants filled out a risk score (online or on paper) to estimate their individual CMD risk. The risk score consisted of seven simple questions about sex, age, smoking status, BMI (height and weight), waist circumference and a family history of premature CVD (age < 65 years) and/or DM2 and calculates the risk to develop a CMD in the next 7 years [14]. The risk score was recently externally validated [15]. High risk was defined as an absolute risk of ≥ 23% for men and ≥ 19% for women. High-risk participants were advised to attend their general practice (second step) for additional risk profiling, including measurement of blood pressure and laboratory tests (e.g. cholesterol levels and fasting glucose). As the last step, patients received tailored lifestyle advice and/or pharmacological treatment.

### Controls

Control participants were invited to complete a health questionnaire including questions about demographic characteristics, CMD risk factors and lifestyle. During the 1-year follow-up, they received care as usual until they were invited for the intervention 1 year later.

### Outcome variables

The primary outcomes of the INTEGRATE study were among others change in CMD risk factors after 1 year. The results on systolic blood pressure (− 2.26 mmHg; 95% CI − 4.01: − 0.51), total cholesterol (− 0.15 mmol/l; 95% CI − 0.23: − 0.07) and smoking (OR 0.75; 95% CI 0.44: 1.28) were used in this analysis. Missing data in the effectiveness analysis were handled using multiple imputation techniques (see Additional file 1 for within trial data and the paper on effectiveness analysis [12]). The long-term cost-effectiveness analysis used the cost per quality-adjusted life year (QALY) as an outcome parameter. A computer-based simulation model was used that included data on disability weights associated with age and disease outcomes related to CMD.

### Measurements

Participants in the intervention group completed the risk score and additional online questionnaires at baseline and 1-year follow-up including among others the EQ-5D-5L measure for health status, work status and absence from work, healthcare costs other than those extracted from the EHR (e.g. costs made for lifestyle interventions or treatment emanating from the program) and non-healthcare costs (participants’ expenses during the study, e.g. travel costs and costs for lifestyle interventions following medical advice).

Participants in the control group filled out the health questionnaire at baseline and additional questionnaires after 1-year follow-up including the same variables as described for the intervention group. Measurements have been described in detail elsewhere [13].

### Data collection

As input for this study, we used the data collected for the effectiveness analysis [12]. In this study, the intervention group included all participants who completed the two-step risk assessment. Because control group participants did not consult their GP, intervention participants were individually matched to participants in the control group based on demographic characteristics to generate the most suitable reference group (details described in Additional file 1).

For both the intervention and the control groups, extracted EHR data were used to establish healthcare utilization during the 1-year follow-up. For the intervention group, EHR data on systolic blood pressure and total cholesterol were collected at baseline (at the first visit to the general practitioner (GP)) and after 1-year follow-up. Case report forms (CRF) and questionnaires were used to collect data on referrals to lifestyle services outside the GP practice.

### Cost data

Cost data were based on EHR, CRF and questionnaire data and on a fixed cost for selection and invitation of the participants per practice. These fixed costs were observed from the clinical study. Table 2.1 in Additional file 2 shows the specification of cost types and their sources. Table 2.2 in Additional file 2 shows the unit prices for different types of costs used throughout this study [16]. Within trial data were collected from a societal perspective, meaning that in addition to healthcare costs, also data on patient and family costs and productivity costs were collected. Cost price year was 2014, the year in which we collected patient data. Unit costs were indexed using Dutch consumer price indices as recommended by the Dutch costing guidelines [17]. Other types of healthcare use outside the GP practice, such as lifestyle interventions for smoking cessation, increasing physical activity, weight reduction, lowering alcohol consumption and improving nutrition were based on CRF and self-report of patients. The patient questionnaires also included data on patient costs. These included costs for travelling, laboratory tests, medication, consultation of other (non-reimbursed) healthcare professionals, subscriptions (e.g. for fitness centre) and other non-specified costs related to the intervention. Finally, data on productivity losses, either from absence of work or from being less productive at work, were based on patient completion of the iMTA Productivity Cost Questionnaire (iPCQ) [18] which was included in the questionnaires.

Intervention costs from a societal perspective consisted of selection and invitation costs, lifestyle intervention costs, healthcare costs and patient costs. All costs were calculated separately for the control and intervention groups.

### The RIVM chronic disease model

The National Institute for Public Health and the Environment (RIVM) chronic disease model (model) is a Markov-type multistate-transition model simulating the evolution of chronic diseases in relation to risk factor levels in the Dutch population. It was developed by the RIVM and applied in multiple cost-effectiveness studies [19–23]. Among other common chronic diseases, it includes congestive heart failure, DM2, myocardial infarction and stroke. Blood pressure and cholesterol are two of the model’s lifestyle-related risk factors. The model describes demography, risk factor prevalence, disease incidence, mortality and their development over time in 1-year steps. These developments depend on transitions between risk factor levels, with subsequent influence on disease incidence and mortality. Systolic blood pressure and total cholesterol both stratify into eight classes in the model. For a more detailed description, see Additional file 3. Relative risks associated with different risk factor levels were derived from literature, whereas incidence, prevalence, transition rates and mortality rates in the model apply to the Dutch population. Disease prevalence is associated with average annual, per patient, costs and with disability weights, reflecting the burden of disease on the individual level. Healthcare costs were based on Dutch costs-of-illness studies [24, 25], and QALYs were computed using the Global and Dutch burden of disease studies [26–29]. Healthcare costs include costs in life years gained. The model takes a healthcare perspective and therefore cannot simulate productivity losses. The model allows specifying alternative scenarios, by adjusting the input parameters. In this study, we simulated two scenarios for the study population: the reference scenario without the observed changes in cholesterol and blood pressure, and the intervention scenario with the age- and sex-specific observed effects, and compared the results.

### Analysis

Costs and benefits after the 1-year follow-up were tabulated (see Table 1 and Additional files 1 and 2). In the long-term CEA, we adopted a healthcare perspective, for reasons described in the previous paragraph. Therefore, only cost data from a healthcare perspective were included in these analyses. In the long-term CEA, the total costs in the first year were selection and invitation costs, lifestyle intervention costs and healthcare costs, and the total costs in later years were the healthcare costs simulated with the model. The CEA was modelled with a one-off INTEGRATE intervention in the Dutch population aged 45–70.

**Table 1:** Costs in control and intervention groups (Euro)

	Control group				Intervention group			
	Mean costs	SD costs	***N*** costs	Average costs per patient	Mean costs	SD costs	***N*** costs	Average costs per patient
Intervention costs^1^	–	–	–	–	–	–	–	–
Healthcare costs (reimbursed)	–	–	–	–	–	–	–	–
Claimed (GP practice) consultations	186.9	196	693	133.94	284.12	237.29	833	244.75
Lifestyle program costs (reimbursed)	–	–	–	–	184.5	391.57	108	20.61
Smoking cessation costs	–	–	–	–	207.14	176.47	8	1.71
Increasing physical activity costs	–	–	–	–	130.8	139.94	44	5.95
Losing weight costs	–	–	–	–	241.37	556.88	44	10.98
Lowering alcohol consumption costs	–	–	–	–	184.56	–	1	0.19
Improving nutrition costs	–	–	–	–	34.88	52.15	49	1.77
Patient costs related to the intervention (not reimbursed)^2^	–	–	–	–	244.38	281.94	360	90.98
Travelling	–	–	–	–	27.62	58.55	52	1.49
Laboratory tests	–	–	–	–	55.99	63.39	168	9.73
Medication	–	–	–	–	151.53	195.88	174	27.27
Other (not reimbursed) healthcare professionals	–	–	–	–	206.92	196.42	61	13.05
Subscriptions (e.g. fitness centre)	–	–	–	–	258.01	204.48	120	32.02
Others	–	–	–	–	231.81	266.6	31	7.43
Other costs	–	–	–	–	–	–	–	–
Productivity costs^2^	10,563.26	13,171.19	6	65.54	12,434.68	10,426.52	8	102.87
	–	–	–	–	–	–	–	–
Total costs	277.96	1495.97	694	199.48	499.17	1583.72	890	459.42

NB Patients in the control group were not asked for costs emanating from the program that could only occur in the intervention group

^1^The intervention costs in this table do not include the implementation costs as these could not be specified to either control or intervention group

^2^The table shows all cost data that were collected in the clinical study (societal perspective). However, as the long-term CEA was performed from a healthcare perspective, patient and productivity costs were not included in this analysis

The change in systolic blood pressure and total cholesterol specified to age, sex and control/intervention group in the 1-year intervention period was calculated and used as input for the model [19, 30] to simulate future healthcare costs and effects on CVD incidence and prevalence, and effects on mortality (see Additional file 3). The time horizon of the simulations was 60 years, representing the maximum lifetime of a cohort starting at the age of 45, the minimum age of the study population. The discount rate for costs was 4% and for effects 1.5%, following Dutch guidelines for cost-effectiveness analysis [31]. To estimate results for the whole of the Netherlands, we multiplied the results for the INTEGRATE population by a factor of 5000/37, as there are around 5000 GP practices in the Netherlands, of which 37 representative practices were included in the INTEGRATE study.

### Scenario analyses

Scenario analyses were performed for the effect sizes on blood pressure and cholesterol levels, selection and invitation costs, and lifestyle + healthcare costs, with parameter values of 80% and 120% of the baseline value. The minimal and maximal changes of the mean systolic blood pressure and total cholesterol levels after 1000 bootstraps were all within this 20% range. The effectiveness study showed a (non-significant) potential effect on smoking cessation. Of the intervention group, 3.25% quitted smoking compared to 2.19% of the control group (OR 0.75; 95% CI 0.44: 1.28) (see Table 1.3 Additional file 1) [12]. It is well known that smoking cessation interventions have highly favourable ICERs [32]. To explore the potential additional effect of reduced smoking, we performed a scenario analysis wherein more patients would quit smoking.

## Results

### Cost and benefits after 1 year

In Table 1 and additional files 1 and 2, details about costs and benefits after the 1-year follow-up are described. The control and the intervention groups both consisted of 967 patients. Baseline characteristics are shown in Table 1.1 of Additional file 1. No meaningful changes were found in EQ 5D-based quality of life between the intervention and control groups after the 1-year follow-up ((− 0.0154; 95% CI − 0.029: 0.004) see Table 1.4, Additional file 1). Table 1 shows the costs in the control and intervention groups. Healthcare costs formed the larger part of the intervention costs. The average total costs in the intervention group were 260 Euro higher than in the control group. As anticipated, the intervention was not cost-effective after the 1-year follow-up, as the intervention was more costly and not effective in terms of quality of life compared to the control group.

### Long-term CEA

**Fig. 1:**
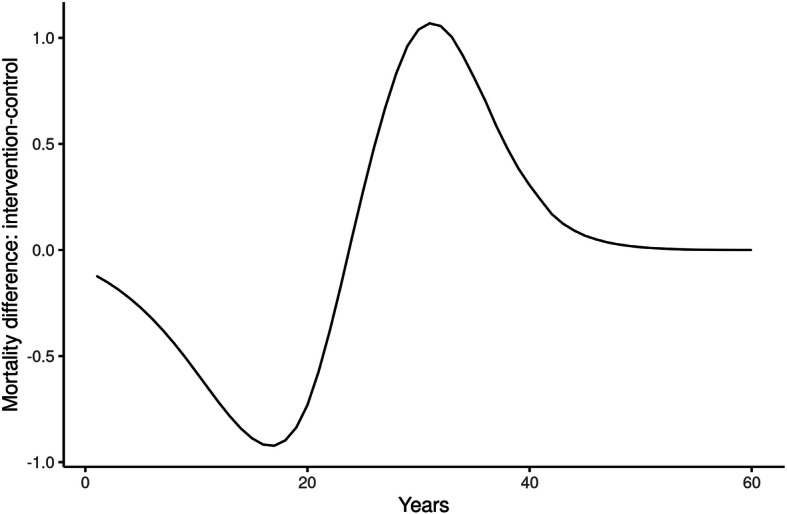
Mortality difference over the years between the control and intervention groups

The intervention resulted in significant improvements in cholesterol and blood pressure (see Table 1.2 in Additional file 1) [12]. These improvements were modelled using transition probabilities as reported in Additional file 3. QALYs were gained via reduced incidence of CVD as shown in Table 2. Table 2 shows the cumulative discounted results after 5, 10, 20 and 60 simulated years. The intervention costs used in the long-term CEA (taking a healthcare perspective, see the “Methods” section) were the difference in total costs minus the difference in productivity costs and minus patient costs: 132 Euro. The ICER of 306,000 Euro/QALY after 60 years indicates that the intervention is by no means cost-effective. The simulations of the intervention scenario showed reduced and delayed morbidity and mortality but not enough to balance the intervention costs that form the majority of the total costs. The disease figures show the difference in (discounted) patient years with the disease. Figure 1 shows the difference in mortality between the intervention group and the control group and demonstrates the delay of death caused by the intervention. In the first decades, the intervention resulted in lower total mortality compared to the control group. After about 25 years, total mortality in the intervention group was higher than in the control group. By that time—due to the intervention—less people had died in the intervention group and thus contributed to the larger pool of possible deaths. On average, patients in the intervention group increased their life expectancy with 1/3rd day.

**Table 2:** Cumulative discounted simulated results for the Netherlands from the healthcare perspective: years with disease, costs, QALYs gained and ICER

	5 years	10 years	20 years	60 years
AMI prevalence	− 9.12	− 39.3	− 121	− 160
CVA prevalence	− 2.82	− 13.7	− 50.1	− 72.4
CHF prevalence	− 1.13	− 5.96	− 27.7	− 46.3
AMI incidence	− 5.52	− 11.4	− 19.4	− 21.2
CVA incidence	− 1.95	− 4.79	− 10.6	− 13.2
CHF incidence	− 0.742	− 2.21	− 7.01	− 9.77
QALYs gained	4.19	19.4	74.0	135
Intervention costs				
Selection and invitation costs: 1200 Euro per GP practice (million Euro)	6.00	6.00	6.00	6.00
Lifestyle + healthcare costs in the year of intervention: 132 Euro per patient (million Euro)	34.4	34.4	34.4	34.4
Future healthcare costs (million Euro)	− 0.0316	− 0.110	0.0548	0.984
Total costs (million Euro)	40.4	40.3	40.5	41.4
ICER (million Euro/QALY)	9.63	2.08	0.547	0.306

*AMI* acute myocardial infarction, *CVA* stroke*, CHF* congestive heart failure, *QALY* quality-adjusted life year gained, *ICER* incremental cost-effectiveness ratio

**Fig. 2:**
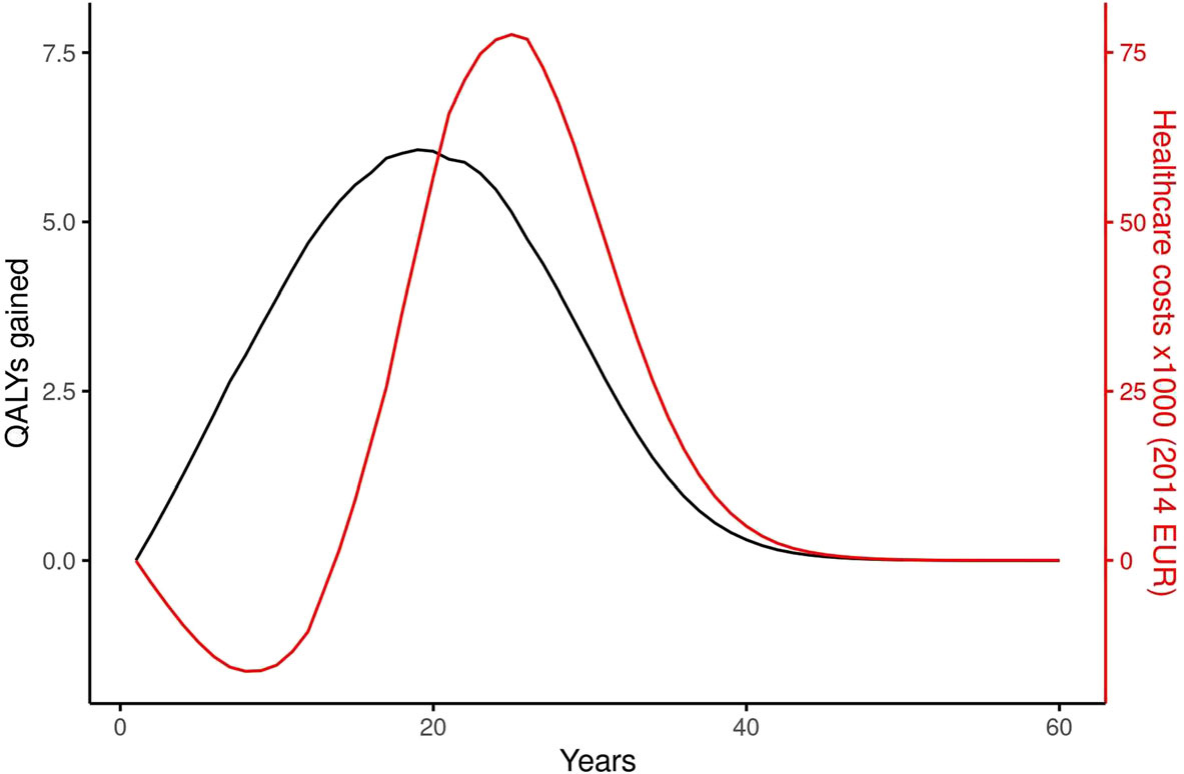
Discounted results over 60 simulated years from the chronic diseases model. Black line: annual number of QALYs gained (1.5% discount rate); red line: annual healthcare costs (2014 Euro, 4% discount rate)

Figure 2 shows the development over time of healthcare costs and QALYs gained in the intervention scenario compared to the reference scenario. QALYs gained were always positive, with a peak after 20 years. In the first 15 years, savings in healthcare costs were anticipated. In the next 5 years however, these savings were nullified and after 20 years, the cumulative healthcare costs were consistently higher in the intervention scenario than in the reference scenario, because of the inclusion of costs in life years gained.

### Scenario analyses

With this ICER far above the cost-effectiveness threshold of 20,000 Euro/QALY that is often used in the Netherlands, none of the scenario analyses showed any result approaching this threshold (see Additional file 4). The scenario analysis on smoking cessation was aimed at exploring a hypothetical maximum health effect of the intervention, i.e. to explore what the yield of the lifestyle improving programs would have been if all smoking patients had successfully participated in a smoking cessation intervention rather than part of all patients following a plethora of other lifestyle improvement programs. This scenario analysis was based on the notion that of all possible changes in lifestyle, smoking cessation is associated with the highest health gains overall. If all smokers in the intervention group (16.55%, *N* = 161) [12] would have quit smoking, the ICER would have been 8000 Euro per QALY. This includes additional lifetime healthcare costs per quitter of 3850 Euro and 0.67 QALY gained per quitter (calculated from Over et al. NTR 2014 [33]). To reach an ICER of 20,000 Euro per QALY, an additional 18 smokers in the intervention group should have quit smoking besides the 31 who already reported to have quit smoking (these effects were not included in the main analysis because they were not significant).

## Discussion

### Summary of results

Although the INTEGRATE study—a large-scale, population-based trial—demonstrated that implementation of a structured stepwise CMD prevention program resulted in a significant decrease in blood pressure and cholesterol levels in high-risk individuals [12], this appeared by no means cost-effective on long-term endpoints. In parallel, the additional scenario analyses showed that cost-effectiveness cannot be achieved even with better treatment compliance and lower intervention costs.

### Strengths and limitations

To our knowledge, this is the first economic evaluation that considers the cost-effectiveness of early detection of CMD with a stepwise approach. Moreover, this is one of the few large-scale clinical studies that investigated the cost-effectiveness of targeted CMD prevention in primary care alongside a trial to ensure that appropriate outcome and cost data were collected. The second strength is that changes in systolic blood pressure and cholesterol levels were modelled simultaneously, because various CMD risk factors are known to have a multiplicative effect [34].

However, also some limitations should be addressed.

For the long-term cost-effectiveness analysis, we adopted a healthcare perspective, because the model cannot simulate costs other than healthcare costs up to now. Taking a societal perspective (including broader societal costs and consequences of the intervention such as productivity losses) could have slightly improved the ICER if positive changes in lifestyle had been achieved. Unfortunately, the effectiveness analysis did not show such an effect [12]. However, benefits of the intervention in terms of prevented CMD events are expected in the future and therefore will not be reflected in productivity costs.

A one-off intervention would not be the “real-world” scenario to implement a preventive intervention. A one-off intervention is most likely more efficient in terms of organization, as fixed costs are distributed over larger groups of patients. In a real-world scenario, the ICER would probably be even higher than our current estimate.

Besides blood pressure and cholesterol levels, no other risk factors were modelled, possibly leading to an underestimation of the reported results. Particularly, the non-inclusion of the non-significant effect that the intervention had on quitting smoking may have contributed to the high ICER that was found. In addition, systolic blood pressure and total cholesterol levels were modelled via discrete classes. The real distribution of these parameters is continuous and even slight improvements in these risk factors will have a favourable outcome on health. Nevertheless, our data from the clinical study also showed substantial numbers of patients with increased levels of blood pressure and cholesterol in the first year after the intervention (Additional file 3). The unfavourable ICERs certainly relate to the fact that the pattern of improvement over patients in the intervention group was not visible in all participating patients. Using discrete levels implies a simplified reflection of the reality; however, the classes used correspond to relative risks based on the best literature available. Furthermore, the model is based on assumptions about the long-term maintenance and changes in risk factors, which could have resulted in a slight under- or overestimation of the long-term outcomes of the intervention. Despite this unavoidable variability, this could not have compromised our main conclusions as only very small health gains were achieved at relatively high costs.

In addition, the model is about 10 years old and the most recent clinical developments in the treatment of diabetes and cardiovascular care have not been incorporated; however, we believe as only small effects were achieved with our intervention these developments could not have been of major influence on our results.

We carefully assessed healthcare costs based on extracted EHR data; however, the EHR contains no data on the use of hospital care. Because the intervention might have led to some hospital referrals, this could have resulted in a slight underestimation of the intervention-related healthcare costs. On the other hand, we might have overestimated the intervention-related patient costs. For example, costs for all new gym subscriptions were considered to be emanating from the intervention. However, we believe that such costs were also made during care as usual.

One final limitation is that the data on patient and family costs and productivity losses were based on self-report. Self-reported data are vulnerable to recall bias. This bias was assumed equal between the intervention and control groups.

### Comparison with existing literature and interpretation of results

The advantage of a stepwise screening approach is that only high-risk individuals are targeted—those who are expected to benefit most from preventive treatment—and therefore assumed to be more cost-effective compared to whole population screening [35]. This is in line with the 2016 guidelines of the European Society of Cardiology which consider targeted CVD screening in high-risk individuals [4].

Modelling studies have demonstrated that targeted prevention strategies for CVD or DM2 in high-risk individuals are most likely cost-effective [21, 36–40]; however, none of these studies evaluated early detection strategies for CMD (CVD, DM2 and CKD). In addition, most of these studies did not include a control group, which is a risk for overrating economic value, as usual care is associated with better healthcare outcomes than no treatment. Furthermore, simulation modelling studies with a positive cost/benefit ratio generally assume lower intervention costs, higher uptake rates, larger treatment effects and sustained compliance than found in clinical trials [11]. Nevertheless, economic modelling of clinical trial data remains very important to project results beyond trial duration to estimate its costs and cost-effectiveness, as follow-up in clinical trials in general is too short to observe subsequent disease incidence and mortality.

Despite promising results regarding lifestyle improvement, another large trial investigating the cost-effectiveness of a European prevention program in primary care focusing on CVD only also demonstrated not to be cost-effective [41]. Although many economic evaluations have been performed in the field of CMD, none of these studies assessed a prevention program for the combination of these diseases [10]. Therefore, it remains difficult to directly compare our results with international equivalents.

The scenario analyses showed that it is very hard to reach cost-effectiveness with the evaluated program. Reducing overall intervention costs with 95% (as shown in Additional file 4) to around €7 compared to about €132 as shown in the INTEGRATE trial is not a realistic goal. Non-response is a designated pitfall of stepwise screening programs, as lack of compliance in different steps might reduce cost-effectiveness. However, optimizing response rates in our study would not have resulted in a cost-effective program, due to relatively high intervention costs.

The most promising way to optimize cost-effectiveness is to introduce more effective lifestyle interventions, especially focusing on smoking cessation. The results of our trial showed no significant effect on smoking [12]. In absolute numbers, 31 of 161 smokers quitted in the intervention group versus 21 out of 161 in the control group. To achieve cost-effectiveness (ICER €20,000 per QALY gained), an additional 18 smokers in the intervention group should have quitted, requiring about three times the effect that was achieved with the current program [12].

In recent years, there has been more attention for the prevention and treatment of CMD in clinical practice due to renewed guidelines and chronic disease management programs. Ongoing individual case finding might lead to a lower prevalence of undetected high-risk individuals for CMD over time [42]. Because individual case finding- and structured early detection strategies are fishing in the same waters, this trend may dilute the detection rate of the program and subsequently reduces its cost-effectiveness.

### Implications for clinical practice and further research

Stepwise CMD prevention in primary care followed by subsequent treatment appeared not cost-effective. Future research should focus on effective lifestyle interventions and the long-term maintenance of its health benefits, especially focusing on smoking cessation interventions.

It is generally assumed that the higher the risk in patients, the more favourable the cost-effectiveness of preventive procedures. Therefore, alternative strategies to identify high-risk individuals might be promising. Dalsgaard and colleagues found that opportunistic screening for DM2 during a regular GP consultation stimulated higher attendance rates. In addition, it was argued that people attending GP practices might have a more unfavourable CMD risk profile and therefore are likely to be at higher risk [42]. Ideally, this would lead to the identification of more cases at lower costs. However, this hypothesis should be confirmed by future research. Another strategy could be the selection of high-risk individuals based on routine EHR data, improving individual case finding in daily practice.

Alongside targeted and individual case finding approaches, expanding efforts for universal prevention of CMD plays an important role to reduce overall CMD risk in the total population.

Given our results and the fact that CMD prevention programs are already implemented in several countries, it is important that these programs are evaluated to assess whether these are a cost-effective use of resources compared to other interventions to reduce CMD risk.

## Conclusion

Implementation of this primary care-based stepwise CMD prevention program is not cost-effective in the long term. Future research should focus on developing more effective lifestyle interventions, with a special focus on smoking cessation, with sustained health effects at reasonable costs. At this moment, the wide-scale implementation of the stepwise CMD prevention program in primary care cannot be recommended.

## Supplementary Information

**Additional file 1.** Within trial data.

**Additional file 2.** Specification of cost data.

**Additional file 3.** Model’s risk factor cut-off values and corresponding transition probabilities.

**Additional file 4.** Scenario analyses.

## Data Availability

De-identified participant data, study protocol and questionnaires are available upon reasonable request from the corresponding author.

## Abbreviations

BMI: Body mass index
CKD: Chronic kidney disease
CMD: Cardiometabolic diseases
CRF: Case report forms
CVD: Cardiovascular disease
DM2: Diabetes type 2
EHR: Electronic health record
GP: General practitioner
ICER: Incremental cost-effectiveness ratio
iPCQ: iMTA Productivity Cost Questionnaire
QALY: Quality-adjusted life year
RCT: Randomized controlled trial
