# Supplementary material for: Cost-effectiveness of a stepwise cardiometabolic disease prevention program: results of a randomized controlled trial in primary care
Source: BMC Med. 2021 Mar 11;19:57. doi: 10.1186/s12916-021-01933-6 (PMC7948329; doi:10.1186/s12916-021-01933-6)
Supplement: Supplementary file 1 — Additional file 1. Within trial data. [file 12916_2021_1933_MOESM1_ESM.docx]

# Additional file 1 (within trial data)

Flowchart 1.

Increased risk
**n=2,836 (39%)**

Intervention group
**n=16,389**

Increased risk
**n=2,240 (41%)**

Non response
n=1,869

Consultation
**n=967 (34%)**

Control group
**n=14,545**

Risk score
**n=7,310 (45%)**

Health questionnaire
**n=5,887 (40%)**

Invited for INTEGRATE
**n=30,934**

Matched† controls
**n=967 (43%)**

Non response
n=9,079

Non response
n=8,658

Low risk
n=4,474

Low risk
n=3,647

# † Participants of the intervention group were individually matched to participants in the control group with an increased risk based on sex, age (in 5-years categories), smoking status and BMI (<25 or ≥25)

| **Table 1.1** Baseline characteristics of participants | | | |
| --- | --- | --- | --- |
|  | | **Intervention group** | **Control group** |
|  | | N=967 | N= 967 |
|  | |  |  |
| **Demographics** | |  |  |
| Sex (%) | |  |  |
|  | Female | 55.4 | 55.2 |
|  | Male | 44.6 | 44.8 |
| Age (years; mean (SD)) | | 62.8 (5.1) | 63.0 (5.0) |
| **CMD risk factors of risk score** | |  |  |
| Positive CVD family history <65 years (%) | | 40.9 | 37.3 |
| Positive DM2 family history (%) | | 25.9 | 28.4 |
| Current smoker (%) | | 16.6 | 16.6 |
| BMI (mean (SD)) | | 25.9 (3.6) | 26.0 (4.0) |
| Waist circumference (mean (SD)) | | 98.2 (11.8) | 99.0 (10.6) |
| **Additional CMD risk factors** (mean (SD)) | |  |  |
| Systolic blood pressure (mmHg) (n=799) | | 135.6 (18.3) | n/a |
| Diastolic blood pressure (mmHg) (n=770) | | 80.0 (9.9) | n/a |
| Total/HDL cholesterol ratio (n=766) | | 3.9 (1.2) | n/a |
| Total cholesterol (mmol/l) (n=764) | | 5.8 (1.0) | n/a |
| LDL (mmol/l) (n=736) | | 3.7 (0.9) | n/a |
| Fasting glucose (mmol/l) (n=715) | | 5.4 (0.8) | n/a |
| **Quality of Life** (mean (SD)) | |  |  |
| EQ-5D score | | 0.926 (0.1) (n=487) | 0.918 (0.1) (n=963) |
|  | |  |  |
| Abbreviations: CVD=cardiovascular disease, DM2= Diabetes Mellitus, BMI=body mass index, HDL=High-density-lipoprotein, LDL=Low-density-lipoprotein | | | |

| **Table 1.2** Change in Systolic blood pressure and total cholesterol between baseline and 12 months follow-up within the intervention group | | |
| --- | --- | --- |
|  | **Total group** | |
|  | **Beta** | **95% CI** |
|  |  |  |
|  |  |  |
|  | N=967 | |
| Systolic blood pressure (mmHg) | -2.26 | [-4.01;-0.51] |
|  |  |  |
|  | N=967 | |
| Total cholesterol (mmol/l) | -0.15 | [-0.23;-0.07] |
|  |  |  |
| Abbreviations: CMD=cardiometabolic disease, HDL=High-density-lipoprotein, LDL=Low-density-lipoprotein, CVD=cardiovascular disease, ICPC= International Classification of Primary Care, ATC=Anatomical Therapeutic Chemical Classification System | | |

| **Table 1.3** Change in smoking between baseline and 12 months follow-up | | | | |
| --- | --- | --- | --- | --- |
|  | **∆ intervention group** | **∆ control group** | **Multilevel analysis**† | |
|  |  |  | **OR** | **95% CI** |
|  |  |  |  |  |
| Current smoker (%) | -3.25 | -2.19 | 0.75 | [0.44;1.28] |
|  |  |  |  |  |
| † all analyses were corrected for baseline values  Abbreviations: BMI=body mass index | | |  |  |

| **Table 1.4 Difference** in quality of life between baseline and 12 months follow-up | | | | |
| --- | --- | --- | --- | --- |
|  | **∆ intervention group** | **∆ control group** | **Linear model**† | |
|  |  |  | **B** | **95% CI** |
|  |  |  |  |  |
| EQ5D | -0.003 | 0.012 | -0.0154 | [-0.029; 0.004] |
|  |  |  |  |  |
|  |  |  |  |  |
| † all analyses were corrected for baseline values | | |  |  |
