# Supplementary material for: Cost-effectiveness of a stepwise cardiometabolic disease prevention program: results of a randomized controlled trial in primary care
Source: BMC Med. 2021 Mar 11;19:57. doi: 10.1186/s12916-021-01933-6 (PMC7948329; doi:10.1186/s12916-021-01933-6)
Supplement: Supplementary file 2 — Additional file 2. Specification of cost data. [file 12916_2021_1933_MOESM2_ESM.docx]

# Additional file 2

**Table 2.1** **Specification of cost types and their sources.**

| **Cost type** | **Source** |
| --- | --- |
| Intervention costs |  |
| Implementation costs  Patient selection  Invitations  Handling | Fixed (bottom-up) price per practice |
| Lifestyle program costs (reimbursed) |  |
| Smoking cessation costs | CRF/Questionnaires (volumes) |
| Physical activity costs | CRF/Questionnaires (volumes) |
| Losing weight costs | CRF/Questionnaires (volumes) |
| Lowering alcohol consumption costs | CRF/Questionnaires (volumes) |
| Improving nutrition costs | CRF/Questionnaires (volumes) |
| Healthcare costs (reimbursed) |  |
| GP practice consultations | EHR (number) |
| Hospitalization | Questionnaires (costs) |
| Out-of-hours primary care services | Questionnaires (costs) |
| Outpatient clinic | CRF/Questionnaires (costs) |
| Emergency care | Questionnaires (costs) |
| Patient costs (not reimbursed)^1^ |  |
| Travel costs | Questionnaires (costs) |
| Laboratory tests | Questionnaires (costs) |
| Medication | Questionnaires (costs) |
| Other (not reimbursed) healthcare professionals | Questionnaires (costs) |
| Subscriptions (e.g. fitness centre) | Questionnaires (costs) |
| Other | Questionnaires (costs) |
| Other costs^1^ |  |
| Productivity costs | Questionnaires & iPCQ |

^1^ The table shows all cost types that were collected in the clinical study (societal perspective). However, as the long-term CEA was performed from a healthcare perspective, patient and productivity costs were not included in this analysis.

**Table 2.2 Unit costs of program components (in 2014 Euro)**

|  | Unit | Cost | Source |
| --- | --- | --- | --- |
| Flyer with lifestyle advice |  | 0.50 | expert opinion |
| Online lifestyle advice |  | 0.00 | expert opinion |
| GP/ practice nurse | Consultation | 33.00 | Dutch costing guidelines^16^ |
| GP/PN | Home visit | 50.00 | Dutch costing guidelines |
| GP/PN | Telephone consultation | 17.00 | Dutch costing guidelines |
| Mental health care (private) | Consultation | 98.00 | Dutch costing guidelines |
| Mental health care (group) | Consultation | 56.84 | Sobell, Sobell, & Agrawal (2009)^17^ |
| Psychologist | Consultation | 64.00 | Dutch costing guidelines |
| Dietician | Consultation | 58.00 | Lammers & Kok (2012)^18^ |
| Complementary medicine | Consultation | 61.52 | average online tariffs (2019) |
| Out-of-hours primary care services | Consultation | 128.97 | tariff: 134.21 (2019) |
| Outpatient clinic | Consultation | 91.00 | Dutch costing guidelines |
| Emergency room | Visit | 259.00 | Dutch costing guidelines |
| Hospital | Day visit | 476.00 | Dutch costing guidelines |
| Physiotherapist | Consultation | 33.00 | Dutch costing guidelines |
| Physical activity | Gym visit | 5.00 | online tariffs: 20 Euro for 4 weeks |
