# Supplementary material for: Cost-effectiveness of a stepwise cardiometabolic disease prevention program: results of a randomized controlled trial in primary care
Source: BMC Med. 2021 Mar 11;19:57. doi: 10.1186/s12916-021-01933-6 (PMC7948329; doi:10.1186/s12916-021-01933-6)
Supplement: Supplementary file 3 — Additional file 3. Model’s risk factor cut-off values and corresponding transition probabilities. [file 12916_2021_1933_MOESM3_ESM.docx]

# Additional file3

**Table 3.1** Total cholesterol classes in the model:

| Total cholesterol: | <5 | 5-6.5 | 6.5-8 | >8 |
| --- | --- | --- | --- | --- |
| Untreated | Class 1 | Class 2 | Class 3 | Class 4 |
| Treated with medication | Class 5 | Class 6 | Class 7 | Class 8 |

**Table 3.2**Systolic blood pressure classes in the model:

| Systolic blood pressure: | <120 | 120-140 | 140-160 | >160 |
| --- | --- | --- | --- | --- |
| Untreated | Class 1 | Class 2 | Class 3 | Class 4 |
| Treated with medication | Class 5 | Class 6 | Class 7 | Class 8 |

Both total cholesterol and systolic blood pressure are risk factors in the Chronic Disease Model (the model). Both are classified in 8 classes; 4 classes with and 4 classes without medication, as drug treatment reduces the relative risk for occurrence of chronic diseases. Increased levels of total cholesterol and systolic blood pressure are associated with higher relative risks. The cut-off values between the different classes are shown in the tables 3.1 and 3.2

**Table 3.3** Total cholesterol transitions in the first year after enrolling the INTEGRATE intervention (N=967 in intervention group):

|  | **Class 1** | **Class 2** | **Class 3** | **Class 4** | **Class 5** | **Class 6** | **Class 7** | **Class 8** | **Total** |
| --- | --- | --- | --- | --- | --- | --- | --- | --- | --- |
| **Class 1** | 110 | 67 | 2 | 0 | 11 | 2 | 0 | 0 | 193 |
| **Class 2** | 75 | 366 | 70 | 1 | 21 | 5 | 0 | 0 | 538 |
| **Class 3** | 4 | 82 | 98 | 5 | 16 | 10 | 2 | 0 | 217 |
| **Class 4** | 0 | 2 | 7 | 2 | 2 | 3 | 3 | 0 | 19 |
| **Total** | 189 | 517 | 178 | 8 | 51 | 20 | 5 | 0 | 967 |

**Table 3.4** SBP transitions in the first year after enrolling the INTEGRATE intervention (N=967 in intervention group):

|  | **Class 1** | **Class 2** | **Class 3** | **Class 4** | **Class 5** | **Class 6** | **Class 7** | **Class 8** | **Total** |
| --- | --- | --- | --- | --- | --- | --- | --- | --- | --- |
| **Class 1** | 86 | 69 | 16 | 2 | 2 | 1 | 0 | 0 | 175 |
| **Class 2** | 76 | 219 | 96 | 11 | 4 | 10 | 4 | 2 | 422 |
| **Class 3** | 21 | 96 | 91 | 21 | 3 | 22 | 11 | 2 | 268 |
| **Class 4** | 2 | 13 | 28 | 13 | 0 | 16 | 18 | 12 | 102 |
| **total** | 185 | 397 | 232 | 47 | 10 | 48 | 33 | 16 | 967 |

**Table 3.5** Total cholesterol transition probabilities:

|  | **Class 1** | **Class 2** | **Class 3** | **Class 4** | **Class 5** | **Class 6** | **Class 7** | **Class 8** | **Total** |
| --- | --- | --- | --- | --- | --- | --- | --- | --- | --- |
| **Class 1** | 0.570 | 0.347 | 0.013 | 0 | 0.059 | 0.011 | 0 | 0 | 1 |
| **Class 2** | 0.140 | 0.680 | 0.130 | 0.002 | 0.039 | 0.009 | 0 | 0 | 1 |
| **Class 3** | 0.017 | 0.379 | 0.452 | 0.021 | 0.075 | 0.046 | 0.011 | 0 | 1 |
| **Class 4** | 0 | 0.108 | 0.370 | 0.118 | 0.109 | 0.152 | 0.143 | 0 | 1 |

**Table 3.6** SBP transition probabilities:

|  | **Class 1** | **Class 2** | **Class 3** | **Class 4** | **Class 5** | **Class 6** | **Class 7** | **Class 8** | **Total** |
| --- | --- | --- | --- | --- | --- | --- | --- | --- | --- |
| **Class 1** | 0.489 | 0.392 | 0.091 | 0.009 | 0.014 | 0.003 | 0 | 0 | 1 |
| **Class 2** | 0.179 | 0.520 | 0.228 | 0.026 | 0.009 | 0.024 | 0.009 | 0.005 | 1 |
| **Class 3** | 0.080 | 0.360 | 0.340 | 0.080 | 0.013 | 0.081 | 0.041 | 0.007 | 1 |
| **Class 4** | 0.019 | 0.127 | 0.278 | 0.125 | 0.002 | 0.154 | 0.176 | 0.119 | 1 |

In tables 3.3 and 3.4 the transitions between classes of total cholesterol and systolic blood pressure because of the INTEGRATE intervention are shown. For example, there were 19 persons in class 4 of total cholesterol initially. Of those, 2 did not change classes, 7 reduced their level of total cholesterol to class 3, and 2 reduced even more, to class 2. The other 8 received medication, and all of those 8 reduced their level of total cholesterol. Tables 3.5 and 3.6 show the transition probabilities
