# Supplementary material for: Cost-effectiveness of a stepwise cardiometabolic disease prevention program: results of a randomized controlled trial in primary care
Source: BMC Med. 2021 Mar 11;19:57. doi: 10.1186/s12916-021-01933-6 (PMC7948329; doi:10.1186/s12916-021-01933-6)
Supplement: Supplementary file 4 — Additional file 4. Scenario analyses. [file 12916_2021_1933_MOESM4_ESM.docx]

# Additional file 4

Scenario analyses:


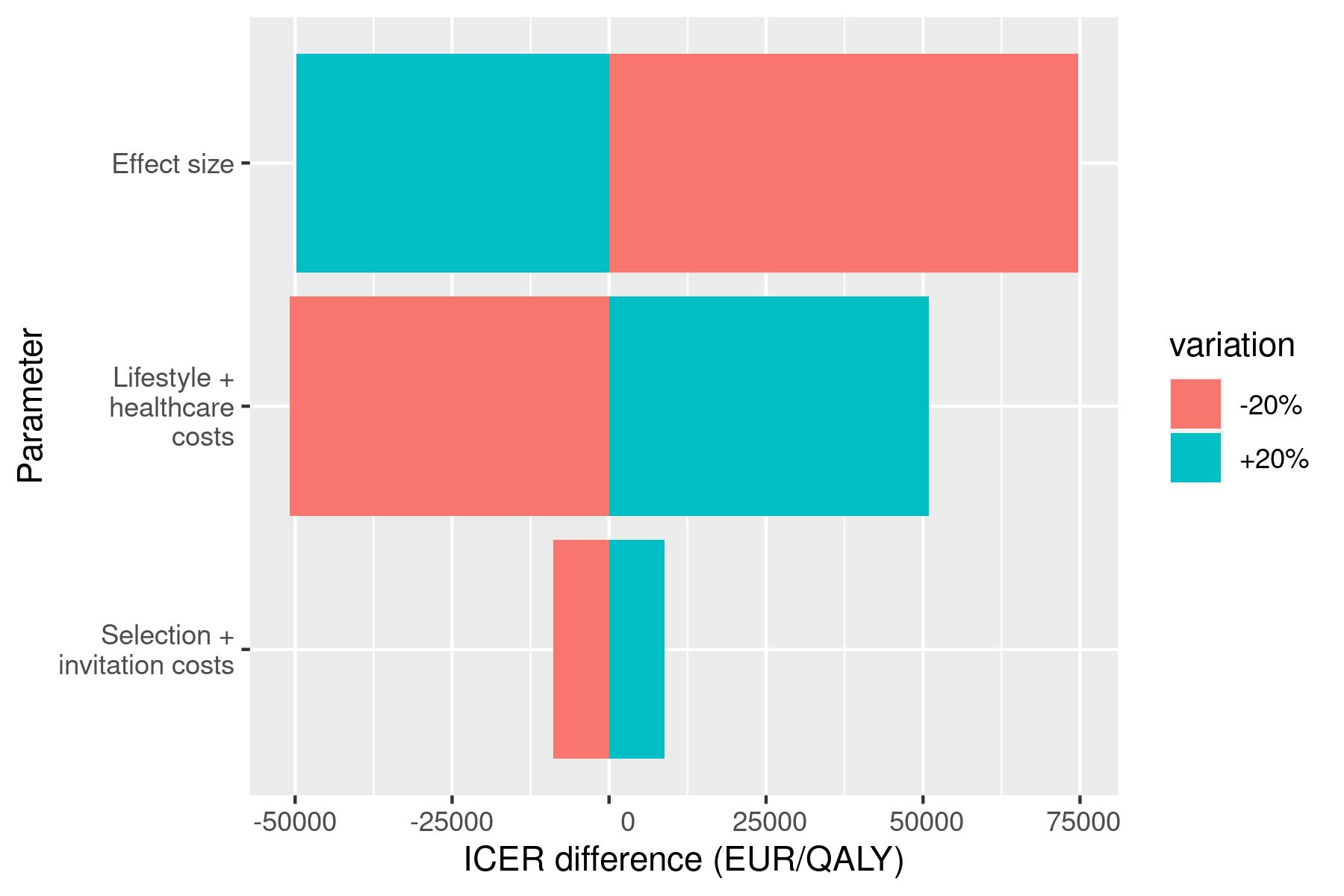


Figure A1. Tornado plot of the difference in Euro/QALY when effect size, selection + invitation costs and lifestyle + healthcare costs were varied with +20% and -20%. Zero corresponds to the default ICER of 306,000 Euro/QALY

Figure A1 shows that decreasing the lifestyle + healthcare cost with 20% would reduce the ICER with around 51,000 Euro/QALY to 255,000 Euro/QALY. The positive effect of increasing the effect size with 20% would be slightly smaller. Variation of the selection + invitation costs would barely affect the ICER.

Cost-effectiveness could hypothetically be achieved when there would be no selection and invitation costs and if lifestyle + healthcare costs would be around 7 Euro instead of 132 Euro (i.e. a reduction of 95%).
